# Experiences of support received by carers of people who are involuntarily admitted to hospital under the Mental Health Act: qualitative study of carers’ perspectives

**DOI:** 10.1192/bjo.2024.44

**Published:** 2024-04-16

**Authors:** Imogen Wells, Abigail G-Medhin, Nicole Owen, Emily L. R. Thelwell, Domenico Giacco

**Affiliations:** Warwick Medical School, University of Warwick, UK; GKT School of Medical Education, King's College London, UK

**Keywords:** Qualitative research, psychosocial interventions, carers, peer support, family members

## Abstract

**Background:**

Carers of people who are involuntarily admitted to hospital report feeling isolated and unsupported by services. The Independent Review of the Mental Health Act (MHA) recommended that carers be supported. However, no research has directly explored what type of support carers would find most helpful when a relative/friend is involuntary admitted.

**Aims:**

To explore carers’ experiences and views around the support they want to receive when their relative/friend is involuntarily admitted under the MHA.

**Method:**

A total of 22 one-to-one interviews with carers were conducted online at three sites across England. Audio recordings of the interviews were transcribed, and data were analysed with thematic analysis.

**Results:**

Four main themes were identified: (a) heterogeneity in the current support for carers, (b) information about mental health and mental health services, (c) continuous support, and (d) peer support and guidance. Carers reported receiving support from professionals, peers and relatives, but this was unstructured, and the extent of support varied across carers. Carers reported wanting more information about mental health services, and for this information to be consistent. Carers also reported wanting emotional support from a single, continuous person, helping them establish a more personal and sincere connection. Peers were also identified as important in the provision of carer support, allowing carers to feel reassured and understood in their experience.

**Conclusions:**

The support received by carers is currently unstructured. To meet the MHA review recommendations, carers of patients who are involuntarily admitted should be allocated a named contact person, ideally with lived experience, to offer information and personal continuity of support.

It is estimated that around 53 337 people have been detained under the Mental Health Act (MHA) between 2021 and 2022.^1^ This has a major impact not only on the lives of those detained under the MHA, but also their family members or friends who support them (‘carers’).^2^ Previous research on the experiences of carers of patients treated under the MHA showed that most felt isolated and unsupported.^2,3^ Carers left without support are at risk of developing mental and physical health conditions.^4^ However, if carers felt supported by services, their caregiving experience may include positive aspects, such as a sense of fulfilment from supporting their family member/friend, enhanced self-esteem and improvements in the relationship with their family member/friend.^5^ Carers can also influence a patient's outcomes. More positive caregiver views around patient treatment were associated with greater symptom improvement among patients.^6^ Improving carer and, consequently, patient outcomes could contribute to substantial economic savings. Currently, carers save the economy £162 billion per year in their support of patients.^7^ Therefore, it is integral that carers receive appropriate support.

## Support during involuntary hospitalisation

Carer support groups (often led by professionals or other carers (‘peers’)) are currently available and have been beneficial for carers.^8^ Studies examining the impact of these support groups focus predominantly on carers of out-patients with severe mental health conditions.^8^ However, the involuntary hospital admission of a loved one can be an extremely distressing and traumatic experience for carers,^3,9^ and the support needs of this carer group may vary significantly from carers of out-patients. There are numerous legal processes for these carers to navigate, depending on a patient's diagnosis, history or sectioning. Additionally, involuntary admission often comes following difficult circumstances and, at times, conflict between patients and their carers. Carers may also experience feelings of guilt surrounding their loved one's detention.^2,3^ As such, there may be a strong need for emotional support during this difficult time. The most recent MHA review has recognised the need to support these carers when a family member/friend is involuntarily admitted to hospital under the MHA, recommending that support be offered to this group.^10^ Therefore, understanding how these carers can be supported effectively is integral. To obtain this understanding, further information from carers about the type of support they want to receive during their family member's/friend's involuntary hospital admission is needed.

Currently, no study has explored the specific support needs of carers of people who have been involuntarily admitted to hospital under the MHA. An increased understanding of carers’ needs and experiences of support could inform the development of a support programme to improve their well-being. To address this gap, the current study aimed to explore: (a) how carers report being supported when their family member/friend is involuntarily admitted to a psychiatric hospital, and (b) how carers think this support could be improved.

## Method

### Design

This was a qualitative, semi-structured interview study using a positivist framework to inform the design and analysis of the study.^11,12^ In line with this framework, a topic guide was used to structure the interviews and patterns in the data were identified through thematic analysis, using the approach developed by Braun and Clarke.^13^ Codes and themes were systematically identified with a hybrid deductive–inductive approach, based on previous literature examining support for carers^14^ and data obtained within the current study.^11,13,15^

The authors assert that all procedures contributing to this work comply with the ethical standards of the relevant national and institutional committees on human experimentation and with the Helsinki Declaration of 1975, as revised in 2008. All procedures involving human patients were approved by the West of Scotland Research Ethics Committee 3 (reference: 21/WS/0098). The Consolidated Criteria for Reporting Qualitative Research (COREQ) guidelines^16^ were used to report on the methods and results of this study.

### Participant recruitment

Participants were eligible to take part in the current study if they were a family member or friend with experience of supporting someone involuntarily treated in a psychiatric hospital within the past 10 years, were aged 18 years or older and had the capacity to consent.

A sample size of around 20 interviews has been suggested as sufficient to achieve thematic data saturation for an interview study using thematic analysis.^17,18^ Therefore, we aimed to recruit a minimum of 20 carers to gain an in-depth understanding of their experience of support during their family member's/friend's involuntary hospital admission and any further support they would have liked during this time. Participants were recruited with a purposive sampling technique,^19^ considering their geographical location (Devon, Coventry and Warwickshire, and East London), gender and ethnic group to obtain diversity in the experiences of support.

Carers were identified through National Health Service (NHS) records and approached by the clinical team at each participating site (Coventry and Warwickshire NHS Partnership Trust (CWPT), East London NHS Foundation Trust (ELFT) and Devon Partnership NHS Trust (DPT)). They were also identified through carer groups and flyers provided in NHS facilities at each site, which asked carers to self-refer for participation. Demographic details of carer recruits were regularly monitored and discussed with both lived experience and professional groups involved in this study, who offered suggestions on ways to obtain a more diverse sample. From these suggestions, clinical staff discussed the study with carer communities frequented by those from typically underrepresented groups (e.g. minority ethnic groups). Lived experience and professional members who were themselves part of an underrepresented group also discussed the study with personal contacts and carer groups they attended. Participants received a brief overview of the study. Those who agreed to take part were contacted by the research team via either telephone or email, to arrange a suitable time for interview. Participants were paid £25 for taking part. Written or verbal informed consent was obtained from all participants. No participants dropped out once they had consented.

### Procedure

All participants were interviewed by the study coordinator (I.W., a female postdoctoral research fellow with a background in health psychology), which they were informed of before their interview. The interviews were conducted online via Microsoft Teams (Microsoft, Redmond, WA, USA; see https://www.microsoft.com/en-gb/microsoft-teams/download-app). Interviews were conducted one-to-one with the participant and I.W. There was no relationship established between I.W. and participants before interview, and no repeat interviews were carried out. Field notes were made by I.W. after each interview, to aid analysis. Transcripts were not returned to participants for comment and/or correction.

A semi-structured topic guide was used to guide the interviews. This topic guide was developed by the research team in co-production with a lived experience advisory panel (LEAP) comprising carers with experience of supporting someone treated under the MHA. These LEAP members also helped to co-produce participant-facing documents. The topic guide asked participants about their experience of receiving support, what support they would have liked during their family member's/friend's involuntary hospital admission and the potential benefits and challenges of carer support.

All interviews were audio-recorded through Microsoft Teams and transcribed verbatim by an external transcription company (Dictate2Us), omitting any personal data. The company respected the same standards of confidentiality used in the University of Warwick and NHS.

### Analysis

In line with the positivist framework,^11,12^ the interview data were analysed systematically with deductive–inductive thematic analysis. A deductive coding framework was generated by a member of the research team (A.G-M., a female research assistant with a background in biomedical science), using findings obtained from previous literature,^14^ with input from I.W. and D.G. (a male professor with a background in psychiatry). The design of this deductive coding framework was also discussed with the LEAP, with feedback being incorporated into the framework by the authors. The transcripts were then systematically coded according to this framework. Each transcript was also coded openly to explore and categorise any additional themes or subthemes found. Interviews were coded independently by four researchers (I.W.; A.G-M.; N.O., a female volunteer researcher with a background in medicine; and E.L.R.T., a female postdoctoral student with a background in psychology) to examine inter-coder reliability. This analysis was facilitated by NVivo version 12.0 for Windows (Lumivero, Denver, CO, USA; see https://lumivero.com/resources/support/getting-started-with-nvivo/download-and-activate-nvivo/). The codebook was refined through several discussions among all authors. Participants did not provide feedback on the codebook. However, six carers from the LEAP were sent the refined codebook and provided further feedback, which was incorporated by authors.^20^ The final codebook represents a formal framework that could be applied to further research examining carer support or toward the development of a carer support programme.^11,12^

## Results

Twenty-two carers across three sites took part in an online one-to-one interview between November 2021 and August 2022 (seven from DPT, seven from CWPT and eight from ELFT). Interviews lasted between 20 and 75 min. Most participants were female (68.1%), White (59%) and a parent of someone involuntarily admitted to a psychiatric hospital (54.5%). The mean age was 50 years. Participant characteristics are summarised in Table 1.

**Table 1:** Summary of participant characteristics

Carers (*n = 22*)	
Age, mean, years (s.d.)	50.4 (14.8)
Gender, *n* (%)	
Female	15 (68.1)
Male	7 (31.8)
Ethnic group, *n* (%)	
White	13 (59.0)
South Asian	6 (27.3)
Black	2 (9.0)
Mixed	1 (4.5)
Relationship to patient being supported, *n* (%)	
Parent	12 (54.5)
Spouse/partner	7 (31.8)
Sibling	2 (9.1)
Cousin	1 (4.5)

### Thematic data analysis

Four overarching themes were identified from the thematic analysis: theme 1, heterogeneity in the current support for carers; theme 2, information about mental health and mental health services; theme 3, continuous support; and theme 4, peer support and guidance. Within each theme are various associated subthemes. An overview of the themes and subthemes identified are outlined in Table 2.

**Table 2:** Overview of themes and subthemes

Theme	Subtheme
1. Heterogeneity in the current support for carers	1.1 Type of support received
	1.2 Impact of support
2. Information about mental health and mental health services	2.1 Information about mental health services
	2.2 Information about patients’ treatment
	2.3 Information to increase understanding and empowerment
	2.4 Named contact for information
3. Continuous support	3.1 Emotional support and reassurance
	3.2 Personal continuity
4. Peer support and guidance	4.1 Peer interaction and support
	4.2 Guidance through the caregiving experience

### Heterogeneity in the current support for carers

Carers described their experience of support during their family member's/friend's involuntary hospital treatment and the impact that this support had on their well-being. The support received appeared to be heterogeneous, coming from various sources, including other family members, professionals and carers with previous experience of supporting someone who had been involuntarily admitted to hospital (‘peers’). Supporting quotes can be found in Table 3.

**Table 3:** Theme 1: heterogeneity in the current support for carers

	Quote	Participant
**Type of support received**		
Quote 1	‘I had to rely on another carer who'd gone through the same and she signposted me, you know, speak to these people or get help from here or this is what they should be doing.’	ELFT01 – parent
Quote 2	‘She [another carer] did [tell] me you can make your complaint and you can speak to the head and you can get … she gets a psychologist, so she gets CBT [cognitive–behavioural therapy] training, all of this stuff she was asking me and I was like, “No”, and she said, “No, you can ask for this, you know.”’	ELFT01 – parent
Quote 3	‘She's [another carer] the one who said to me that I should talk to my daughter's advocate and I didn't even know what the word advocate was.’	DPT05 – parent
Quote 4	‘That's what the mother of this boy said to me – ask to have an interview or a meeting with the doctor even online.’	DPT05 – parent
Quote 5	‘I received information about where the patient would be staying like the estimated duration, who they'd be approached and engaged by, and what the fundamental rights, and the obligations that they have and the fulfilment by staff to sort of be seen upheld, that was the means of information.’	ELFT13 – parent
Quote 6	‘Just some insight as to what the procedures would involve and their [general practitioner's] perspective on why it was necessary and proportionate in their eyes and to the current situation that they were going through.’	ELFT13 – parent
Quote 7	‘She [a social worker] was quite thorough, she also sort of gave us our rights, she said if she was to be detained we could contest, yeah. So, she did what, you know, she informed [us] on what would happen next.’	CWPT04 – parent
Quote 8	‘Family were also there to lean on but friends were there more so in terms of just support and being able to offload the burden.’	CWPT04 – parent
Quote 9	‘Just even taking out the emotional stress of having someone who is acutely unwell and likely to be suicidal, psychotic, whatever.’	DPT02 – parent
Quote 10	‘There was one family member who had similar, went through something similar with her family member, so we could relate to it and that helped just to offload a burden, so just to share that burden with someone.’	CWPT04 – parent
Quote 11	‘I think, yeah, we [participant and their partner's mother] do support each other, I guess, because we do talk very often and not only about him [participant's partner]. I feel I can come to her with anything and I think she feels the same way, so it's really good to know that.’	ELFT03 – partner
Quote 12	‘I've got friends who are kind of in the same boat so we help each other. They help me. If I need to chat to her or them, I will occasionally message them or have chat to say come over or we can meet up and you know just kind of have a chit chat, yeah.’	ELFT02 – parent
Quote 13	‘I know that if something would happen in the future, I know that she [partner's mother] will be there for me and for us, and I would be, you know, I would do the same for her.’	ELFT03 – partner
Quote 14	‘Basically, we didn't feel supported at that time [during their family member's involuntary hospital admission]. We knew she was unstable, but it was difficult because it was a lot of waiting around until someone contacted us. And we had very little communication from the psychiatric ward.’	CWPT04 – parent
Quote 15	‘There was no real contact with any professional, you know, a clinician or psychologist or whoever it might be, a doctor. We just didn't know what to expect when our daughter returned or if she returned. There was no sort of real feedback.’	CWPT04 – parent
Quote 16	‘I can see that people can choose to consent to have their partner or not- next of kin have information and have access to the services, whereas I was offered absolutely nothing. I felt like I didn't know half of the things that I know now, things could have been maybe have been different.’	CWPT09 – partner
Quote 17	‘I've been offered a carer support group I can attend on a Tuesday. But it just doesn't fit in with my diary because of work, so I haven't had the opportunity meeting other carers.’	CWPT04 – parent
Quote 18	‘I know quite a few families that they … they've got children that are autistic for instance, but they would never tell their friends, or they have other people that don't know about this situation because they see it as a … they see it to be very embarrassing.’	ELFT12 – sibling
Quote 19	‘I think they [other carers] feel isolated so they'd rather not tell anyone kind of, they kind of deal with it themselves, which is obviously is not the way, you need to get help.’	ELFT01 – parent
**Impact of support**		
Quote 20	‘I think there was just something comforting about knowing that she understood, really. And I didn't need to explain things. I didn't need to explain how it impacted me. I could just talk about the reality of the situation rather than having to justify how I felt in some way.’	DPT02 – parent
Quote 21	‘I know that I can talk to her and she really understands, and there's no judgment on my son or me as a mother or anything. You know, she gets it and vice versa.’	DPT01 – parent
Quote 22	‘It's very hard for people who haven't got someone they love in this experience to just understand what you're going through, lots of people can't understand why, you know, why they haven't had help sooner or why they're not getting better.’	DPT01 – parent
Quote 23	‘It really felt isolated but joining the carers group, I've realised that I wasn't the only person in the same … you know, I have other people in the same boat as myself. So, it gave me a bit of hope and it gave me a bit of belonging to know that.’	ELFT04 – parent
Quote 24	‘Meeting them [other carers] gave me hope because I thought, oh, there are—here are people that are supporting one another and there is hope for my son and there is life after this, you know, for people, so it was good.’	DPT01 – parent
Quote 25	‘It was because she [another carer] gave me hope that there will be light at the end of the tunnel; there's a start, a middle, and an end.’	CWPT05 – parent
Quote 26	‘So, it's very cathartic. It's very therapeutic because you get to, I don't know, like oh my God, what, this is … I mean, you meet other people, and their problems are million times worse than yours.’	CWPT02 – sibling
Quote 27	‘I don't know whether it's more difficult to have three children who are autistic and have ADHD [attention-deficit hyperactivity disorder] or whether it's more difficult if you have one who doesn't have these problems, I don't know. I just I thought, my God, what am I whining about?’	CWPT02 – sibling
Quote 28	‘There was people [carers in support group] they said, their daughter, because they were high functioning in autism. It was difficult because they could understand calories. Well, my daughter isn't high functioning, so she doesn't understand calories. And it was quite, also, I'm just thinking which was better? Then I said, “You know what? Maybe I'm the lucky one because at least I can feed her.”’	ELFT02 – parent

#### Type of support received

Although there were some similarities in the type of support received across participants, this support appeared to vary with regard to the participant or person providing support (e.g. peers, family members or professionals). Most participants reported receiving some information and advice around their role in the care of their family members/friends during involuntary hospital admission. Some participants had been signposted to relevant services and contacts by peers, which was initially unknown to these participants (Table 3, quotes 1–4).

Several participants also described receiving information from professionals, such as social workers and doctors. This information primarily focused on patient and carer rights, including relevant MHA sections, the treatment process in psychiatric hospitals and a patient's right to a mental health tribunal (Table 3, quotes 5–7).

Some participants reported receiving support regarding their feelings around their family member's/friend's involuntary hospital admission, often provided by their family members, friends or peers. By communicating with these people, participants felt able to offload the burden felt around caring for their family member/friend (Table 3, quotes 8–10).

In some cases, participants also discussed matters unrelated to their family member/friend, with the reassurance that someone was available to talk when needed (Table 3, quotes 11–13).

However, a few participants did not receive support during their family member's/friend's involuntary admission. Some participants reported that they were not offered support (Table 3, quotes 14–16), or that the support offered did not fit their schedule, making it impossible to receive (Table 3, quote 17). Others reported not receiving support because of feelings of privacy or shame around their situation (Table 3, quotes 18 and 19).

#### Impact of support

On receiving support, participants described feeling understood and validated in their experience, particularly as a result of peer support. They reported feeling a deep understanding and unspoken bond with peers because they had gone through a similar experience (Table 3, quotes 20–23).

Some participants also felt reassured by peers’ depictions of their more positive experiences of care, such as their family member's/friend's improvement. This reassurance increased participants’ feelings of hope for their own family member's/friend's recovery (Table 3, quotes 24 and 25).

A few participants also described feeling reassured by peers’ negative experiences of care, which appeared to change their outlook on their own situation, feeling that their situation was perhaps not as bad as others (Table 3, quotes 26–28).

### Information about mental health and mental health services

Although most participants did receive support during their family member's/friend's hospital admission, they identified other areas where they would have benefitted from further support. Specifically, participants wanted to receive further information about mental health services, including treatment provided to patients by these services, and for a consistent, named contact to provide this type of information. Supporting quotes can be found in Table 4.

**Table 4:** Theme 2: information about mental health and mental health services

	Quote	Participant
**Information about mental health services**		
Quote 1	‘And then also I think people need to know about carer's rights at work, the right to ask for flexible time, flexible working and paid time off.’	CWPT02 – sibling
Quote 2	‘I think that it helps in the eye of the law to know what the law and what the rights are of your loved one, and what your rights are as the carer, so that you can actually have information when you go in there to say, look, I am the next of kin, I know this, I can have information about their medication.’	CWPT06 – parent
Quote 3	‘The social workers and the carer's assessment … they don't … you go and talk to … they don't tell you exactly what you're entitled to. They wait for you to say what you'd like, you know?’	ELFT06 – parent
Quote 4	‘I think [carers] ought to know about carer's assessment, carer's rights, the effect of working on carer's allowances, there are also issues to do with the benefits that the person you look after gets, they can have those taken away because you get carers allowance, all that sort of thing.’	CWPT02 – sibling
**Information about patients’ treatment**		
Quote 5	‘So I would have liked to have known what they're going to do with him [participant's sibling], what treatment they're going to give him.’	CWPT02 – sibling
Quote 6	‘Just like what I can expect in a couple of weeks or what are the stages of healing and … because at the moment they're saying … because they keep saying that, “Oh, your wife's doing well and she's been really well.”’	CWPT03 – partner
Quote 7	‘There was only so much information they can share outside because she was an adult, and if she hadn't consented to share that information with anybody, they wouldn't tell us anything.’	CWPT04 – parent
Quote 8	‘Or your son's … all right, yeah, no, it's confidentiality. You don't want to know what they've talked about. But they have got to phone up and say, “Your son's came, have come in. We've seen him. How are you?”’	ELFT06 – parent
Quote 9	‘I understand the confidentiality around it. I do understand that there's only so much they can disclose, but this is just basics about someone being well.. you know…what kind of a day she's [participant's daughter] having.’	CWPT04 – parent
Quote 10	‘I think the hospital should understand that if the patient has signed the form once, when they actually talked to their parent or whoever is their carer, the hospital should give some kind of leeway and understand that, to be completely in the dark, when you're supposed to be the person caring for the other person. It makes your life unliveable.’	DPT05 – parent
**Information to increase understanding and empowerment**		
Quote 11	‘I did talk to my mum and dad [about participant's partner], but I did try and keep them sort of not fully in the loop because I just would know that they worry about me because with the stigma that's attached to mental health, people's automatic assumption is that you're going to be murdered by a partner when they're not well and that's the ultimate risk, so I felt like a lot of my friends were worried.’	ELFT03 – partner
Quote 12	‘Well, I didn't know about schizophrenia really apart from what you read in papers and that. I mean you see, the only headlines you get on schizophrenia is people going mad with knives don't you I mean that's the only thing you hear schizophrenia is that everyone's going … killing people.’	CWPT01 – partner
Quote 13	‘It took me a little while as well to accept it [participant's son's disorder], and as well you're looking at the stigma of how it impacts on us as a family and even in the [Jamaican] community. So, that didn't help, the stigma was really, really, really strong. And I used to hide it from friends because nobody understands mental health. You know, in our community, they think somebody *obeah* – *obeah* means something like black magic or something like that, so I didn't want it for him either.’	ELFT04 – parent
Quote 14	‘They [patients] would get stigmatised here [in minority ethnic groups] because they look at future, you know, can they get married, all this kind of stuff, they want to get a job or make friends, you know, people get scared to kind of label that as if you're mad.’	ELFT01 – parent
Quote 15	‘I never knew about like schizophrenia and things like that. I mean how it was affecting her. I did this course with social services and that really opened my eyes in a world where, I'm having a conversation with you and two other people would be talking in me ear at the same time.’	CWPT01 – partner
Quote 16	‘At the end, they were doing some courses that I attended and that kind of helped understanding about eating disorder and there are various, bulimia and other kind of eating disorders so that was nice, yeah.’	ELFT02 – parent
Quote 17	‘I now know why she does certain things or wants to do certain things, and I recognise when things are going wrong, yeah.’	DPT12 – partner
Quote 18	‘When somebody's behaviour alters you're going to wonder why … they don't want to do something, they don't want to get out of the bed in the morning or something; when it's explained to you, oh, that's normal symptoms of the illness, well, that's fair enough. But until then you wonder what's happening.’	DPT12 – partner
Quote 19	‘The particular thing my son had was psychosis, so there's very little out there, you know, information-wise. And obviously, for every person, it's going to be very different, isn't it? So, I would have liked them [professionals] to reach out to speak to me and give me a bit more in-depth knowledge about it, yeah, as to why it happened and how to reduce it happening again, things like that.’	CWPT05 – parent
**Named contact for information**		
Quote 20	‘There wasn't like one place you can go and try and get answers, and some people sort of didn't really know what had happened. You know, you'd get different answers from different people.’	DPT08 – partner
Quote 21	‘But, it's just because there was no real contact with any professional, you know, a clinician or psychologist or whoever it might be, a doctor. We just didn't know what to expect when our daughter returned or if she returned. There was no sort of real feedback.’	CWPT04 – parent
Quote 22	‘The next call I had was from the nurses, doctors, but I wasn't speaking to one person, I was speaking to different people at each time. They were asking me the same set of questions; I was really having to repeat myself.’	CWPT04 – parent
Quote 23	‘The nurses who were on the ward were inconsistent, they were very inconsistent, there was change of rota, staffing, and there was handovers, so you never got the same person, and if you wanted just to touch base on her [participant's daughter's] well-being, it was quite difficult because one member of staff would pass on a message to another member of staff and that person would never get back to you.’	CWPT04 – parent
Quote 24	‘For someone who is very good at laying the … to express things in lay English, to contact the family members to sort of say this is the situation, this is what's happened, this is what's going to happen from this point forth. It would've been desirable.’	ELFT12 – sibling
Quote 25	‘It feels really vital to me that there's clarity, and it wouldn't take, it's not a, it's not rocket science, it's actually down to simple clear understanding of what to expect.’	DPT03 – parent
Quote 26	‘I think that's the thing, just knowing what's going on and hearing it, and hearing it from people that are all saying the same things rather than sort of just hearing bits and bobs from different people.’	DPT08 – partner
Quote 27	‘Wouldn't it be nice is to have an advocate for my son and also an advocate for our family so that there's somebody that could tell me what's happening when it's happening, they don't have to give away confidentiality, but just to know that someone knows our story and we don't have to keep repeating ourselves and explaining who we are, what we are, what's happened.’	DPT01 – parent
Quote 28	‘Maybe the carers should have an advocate and yes, I think that would be wonderful, to have an advocate that can liaise between the carer and the hospital so that the hospital knows what's going on for the carer.’	DPT05 – parent

#### Information about mental health services

Most participants felt that they needed more information about mental health services, including legal information relevant to their family member/friend and to them as a carer (Table 4, quote 1). It was reported that receiving information about their rights could help carers access other important information that was legally available to them (Table 4, quote 2).

Participants also reported needing information about the practical support they could receive during their family member's/friend's treatment, including carer's assessment and carer's allowance. Participants stated that this information is not made readily available to carers currently (Table 4, quote 3). Participants also felt it was important for carers to know about the potential impact of carer's allowance on their work and finances (Table 4, quote 4).

#### Information about patients’ treatment

Most participants reported wanting to receive more information about their family member's/friend's treatment options, how their treatment was going and what to expect during their treatment (Table 4, quotes 5 and 6). However, some participants felt that patient confidentiality was a barrier to the provision of this information (Table 4, quotes 7 and 8). Professionals must maintain patient confidentiality and thus cannot provide certain information to their carers unless patients consent to that information being shared. Most participants were aware of this, but felt that more basic information, such as information on their family member's/friend's general behaviour and well-being during their hospital stay, should be made available to carers without the need for consent (Table 4, quotes 8–10).

#### Information to increase understanding and empowerment

Participants and other family members/friends often had misconceptions about their patient's disorder stemming from a lack of awareness, which was often influenced by mental health stigma (Table 4, quotes 11 and 12). This had an impact on the way they felt toward their patient, and seemed particularly prominent within minority ethnic groups (Table 4, quotes 13 and 14).

Participants who received more information about their family member's/friend's condition reported gaining a better understanding of their loved one's experiences (Table 4, quotes 15–18). Those who did not receive such information reported wanting this information to feel more equipped in supporting their family member/friend by increasing their awareness of potential triggers (Table 4, quote 19).

#### Named contact for information

Participants reported receiving inconsistent or incomplete information about their family member's/friend's hospital treatment ( Table 4, quotes 20 and 21). They also described having to repeat information about their family member/friend to various professionals, noting a supposed lack of communication across the clinical team (Table 4, quotes 22 and 23). This unpredictability around the information communicated caused uncertainty and confusion among participants.

Because of this, participants reported wanting a specific person to share clear, comprehensive information between carers and hospital staff. Having this could ensure that the information received by both parties is accurate (Table 4, quotes 24–26).

Some participants felt that a separate advocate working specifically for carers could help to ensure that accurate information is communicated consistently between carers and the mental health service (Table 4, quotes 27 and 28).

### Continuous support

During their family member's/friend's involuntary treatment, participants reported wanting a formal support service where they can express their emotions. Participants also consistently reported the need for this support to be provided by a single, continuous person. Supporting quotes can be found in Table 5.

**Table 5:** Theme 3: continuous support

	Quote	Participant
**Emotional support and reassurance**		
Quote 1	‘You think you're hopeless and then you're dealing with all these emotions that you don't know where they've come from, and they're hard to deal with.’	CWPT05 – parent
Quote 2	‘You go into it [caring for someone involuntarily hospitalised] blind, it's very distressing, and I still feel emotional now, quite traumatic.’	CWPT05 – parent
Quote 3	‘My concerns, like my concerns aren't taken seriously//like [a professional] will be like oh you know that's just the illness isn't it and you know like quite snarky and I'm like I'm not saying I'm going to abandon her, they just don't … I don't know, they make me feel guilty and feel bad.’	CWPT03 – partner
Quote 4	‘I'd like to see the professional face-to-face. Discuss things and ask things and, you know, that… As I said, there is nothing out there for people going through this, and I'd love them to say, “Look, we got a helpline for you where we've got someone who could speak to you.”’	CWPT05 – parent
Quote 5	‘Just somebody checking in on us just to see how we were coping. I think just more personal contact really other than just when we phone up to find out an update.’	CWPT05 – parent
Quote 6	‘This is a layer that we—they're offering it to you, you may not feel that you want it at this stage but is it okay if I check in with you tomorrow? Is it okay if I check in with you in two days’ time? Would it help if I was able to visit with you and just saw you in the car park for a few minutes before you went home.’	CWPT06 – parent
Quote 7	‘(Sighs) You just want someone to be able to say that it's going to be okay.’	CWPT09 – partner
Quote 8	‘I don't think anyone really realises the extent of damage it's doing to me. And then if I do complain then it's normally, “Oh, suck it up, you're the man.”’	CWPT03 – partner
Quote 9	‘Apparently if you say that [that you're not okay] then you're considered weak and then that kind of makes me not want to talk to anyone.’	CWPT03 – partner
**Personal continuity**		
Quote 10	‘I've spoken to him [a social worker] numerous occasions, but their case loads are so big that I think sometimes they get confused and it can seem a bit impersonal. It would be really nice to know there was somebody who cared, (chuckles) who took a genuine interest, you know, and not—it wasn't just, oh it's just another case.’	DPT01 – parent
Quote 11	‘I think there should be someone in their [mental health service] team who knows who you are and you know who they are, and not just a sort of disembodied voice on the end of the phone that you—and who really knows you.’	DPT01 – parent
Quote 12	‘Just to know that someone knows our story and we don't have to keep repeating ourselves and explaining who we are, what we are, what's happened.’	DPT01 – parent
Quote 13	‘A confidential adviser or a buddy assistant to talk to over the phone or in a private safe room, one-to-one, not in coffee mornings but like regular get-togethers or sessions to just connectively feel part of the same community and environment.’	ELFT13 – parent

#### Emotional support and reassurance

Participants described the experience of their family member's/friend's involuntarily hospital admission as distressing and traumatic (Table 5, quotes 1 and 2). Although some participants reported receiving emotional support during their family member's/friend's involuntary admission, others described not receiving this type of support at all (Table 5, quote 3). As a result, they reported needing a support service where they could receive regular check-ins and reassurance (Table 5, quotes 4–7).

Any support service offered to carers needs to be non-judgemental. One participant reported being judged for sharing his feelings because of his gender, which had a detrimental impact on his desire to share these feelings in the future (Table 5, quotes 8 and 9).

#### Personal continuity

During their family member's/friend's involuntary admission, participants reported being contacted by various professionals about their family member/friend in a way that felt impersonal. Participants described feeling like another ‘case’ to professionals rather than a person who is struggling (Table 5, quote 10).

Because of this, participants reported wanting to receive support from a single, consistent person (Table 5, quotes 11 and 12). Participants reported that having this single person to provide support could help them to develop a relationship that felt sincere, personal and connected (Table 5, quote 13).

### Peer support and guidance

Participants consistently communicated that carers should be in contact with peers during their family member's/friend's involuntary psychiatric admission. They reported needing support and guidance from peers because of the knowledge these peers have likely obtained from their previous experience. Supporting quotes can be found in Table 6.

**Table 6:** Theme 4: peer support and guidance

	Quote	**Participant**
**Peer interaction and support**		
Quote 1	‘It can be a very lonely place. It could be very isolating. Not something you want to speak to people about when you go to work because they wouldn't understand what you're going through anyway.’	CWPT05 – parent
Quote 2	‘It's a very lonely path to walk when you have a child who's as ill as mine has been.’	DPT02 – parent
Quote 3	‘It's just it will be nice … I mean, it won't be nice but it will be nice to know talk to the people going through it because at the moment it just feels really lonely.’	CWPT03 – partner
Quote 4	‘To give people something that … somebody who understands them. That may not be able to help them but can listen and totally understand where they're coming from.’	CWPT05 – parent
Quote 5	‘It's like an organic connection between the two because you've got that shared trauma that no one else can understand.’	CWPT09 – partner
Quote 6	‘A feeling that somebody actually knows the stress and anxiety and the trauma that the families go through so they're not … you know, so they understand, like they really understand and they understand that actually, you might be quite tired or you might not be thinking straight yourself at the time and you just need support, someone alongside you.’	DPT01 – partner
Quote 7	‘I think that it's finding out from the person who is the carer of what their relationship is with their loved one, what, you know, to say that I've been in this position is to take a certain level of honesty from the peer mentor to say how they managed to have experience of the … under the section, being sectioned under the Mental Health Act, what happened, and for me, I think it's trying to see that there's hope at the end.’	CWPT06 – parent
Quote 8	‘I think it's very, very important for carers to have a support network, like a support group or someone to talk to. And that they know, at least that even though if they don't have the need but they know where they can call or what they can do if they feel the need to, or if they need any kind of help or if they're unsure of certain things.’	ELFT03 – partner
Quote 9	‘A professional's been educated to know those things but a carer would have gone through it themselves personally. You can't learn everything out of a textbook, sometimes real-life events give you better knowledge.’	CWPT05 – parent
Quote 10	‘To have someone that's got that experience and actually having had it themselves, no amount of learning is ever going to match that. It's something else that you can't … I don't know. If people, I suppose, go to … go to war and stuff like that, they … the people that have been there, if they've got like an immediate collaboration, you see people from the Forces, they meet each other and they talk about their experience, they've just got an immediate sort of connection that you can't force.’	CWPT09 – partner
Quote 11	‘I think that it will be medical advice, plus the patient, plus the human touch [that peers can provide to carers] equals positive progress.’	DPT06 – cousin
Quote 12	‘I think people who understand what it's like to be so impotent in being able to do anything, particularly mothers I think, it probably applies to mothers more than anybody, to help you to be able to detach, to be able to go off and do something that's good for you.’	DPT03 – parent
Quote 13	‘I was hoping to meet a lot more South Asian people that were very dis—well, they weren't discreet because they attended the meeting but they were initially sort of trying to not do something about it.’	ELFT12 – sibling
**Guidance through the caregiving experience**		
Quote 14	‘You're first going in and coming out and just crying your eyes out because you've gone into a building that you're very alienated and never been, been to a general hospital. You've never been to a mental health hospital.’	ELFT06 – parent
Quote 15	‘Family situations are different, and relationships are different. But I think again, it's knowing that first admission is petrifying, it's a terror, I feel.’	DPT03 – parent
Quote 16	‘I think back to how completely out of my depth I was when all this started and how much I've learned, how nice it would have been to just have someone to walk that path with me a little bit, and walk alongside me and hold my hand every now and again, just kind of point you in the right direction.’	DPT02 – parent
Quote 17	‘Someone who understood but, more than that, someone who could help you navigate the complexities of having someone under the Mental Health Act.’	DPT02 – parent
Quote 18	‘I didn't need advice, I needed somebody to help me navigate a system I had no knowledge of.’	DPT03 – parent
Quote 19	‘I'm not expecting them to know everything about carers rights and Carers Act, but it's just an empowering thing, it just enables them to understand, you know, what it's all about to be a carer.’	ELFT04 – parent

#### Peer interaction and support

Both participants who had and had not received support from peers during their family member's/friend's involuntary admission highlighted the need for all carers to receive this type of support. Participants reported feeling isolated during their experience, primarily because of a lack of understanding from others (Table 6, quotes 1 and 2), and so wanted someone who could understand their experience whom they could connect with (Table 6, quotes 3–6). Participants wanted to feel reassured by those who had been through a similar experience, and to understand the coping strategies peers used during their family member's/friend's treatment to inform their own (Table 6, quotes 7 and 8).

The level of understanding that a peer can offer through their experience of supporting someone who has been involuntary admitted to hospital was highly valuable to participants, and was seen as additional to professional support (Table 6, quotes 9–11).

Similarities between carers was also noted as an important consideration for peer support. One participant noted that having a similar relationship with a family member/friend was important for mutual understanding between peers (Table 6, quote 12). Another participant discussed wanting more people from a South Asian background in attendance at group meetings, particularly those who also felt some level of privacy around their family member's/friend's admission (Table 6, quote 13).

#### Guidance through the caregiving experience

Participants also reported wanting guidance around the mental healthcare system from those who have been through the system before. Although this was highlighted as something that could be useful for any carer, particular importance was placed on those whose family member/friend had been involuntarily admitted for the first time, as participants noted how distressing the first-time experience can be (Table 6, quotes 14 and 15).

Participants also noted how the complexities surrounding involuntary hospital admission can feel extremely overwhelming for those dealing with a first admission, and how it would be useful to have someone with that knowledge to provide some direction (Table 6, quotes 16–18).

One participant also highlighted how empowering it may be for carers to learn how to navigate the mental healthcare system from those who have inside knowledge, allowing them to gain more understanding of the caregiver role (Table 6, quote 19).

## Discussion

### Summary of findings

Carers of people treated under the MHA report that the support received during admission is generally unstructured and inconsistent, with some carers receiving no support at all. Carers identified three simple needs that, if addressed, could improve the current support offered to this group. The first is the need for carers to receive more information about mental health services, including the treatment provided within these services, and for this to be clearly communicated via a named contact. The second is the need for personal continuity in the delivery of emotional support to allow carers to feel a sincere connection and sense of comfort in confiding about their feelings. The third need is for carers to receive peer support to help them feel reassured and understood in their experiences.

### Comparison with literature

Carers reported the need for continuity around both an information and support contact during their family member's/friend's involuntary hospital admission. The need for continuity regarding support aligns with previous qualitative research where patients and clinicians reported that personal continuity of care could improve the quality of the relationship with the clinician and enhance holistic care.^21^ Challenges to this may be posed by the fact that often information about a carer's patient under treatment is provided through multidisciplinary teams (MDT), making it difficult for carers to receive information from a consistent contact, as each professional is likely to know different information depending on their speciality.^22,23^ One solution for this may be to have a single person attend MDT meetings (carers in the current study suggest a carer advocate) and feed back the information to carers.

The feeling of isolation carers reported in the current study reflects previous qualitative findings examining carers’ experiences of their family member's/friend's involuntary hospital admission.^2,3^ Peer support may be one avenue to address this feeling of isolation, with carers in the current study reporting wanting support from people with similar experiences. The ability to interact and receive support from peers was found to be highly beneficial in studies examining patients with an acute episode of mental illness.^24^ Previous research also highlighted the benefit of carer support groups, reflecting carers’ feelings toward this type of support in the current study.^8^ The social identity theory may help to understand these findings, positing that a sense of belonging to a group can have a positive influence on an individual's self-esteem.^25^

The need for emotional support highlighted by carers in the current study aligned with previous qualitative findings from the nearest relatives of people who have been involuntarily admitted to hospital under the MHA (a family member who holds specific responsibilities and power for someone detained under the MHA).^9^ Both studies suggest that this support be offered more widely to carers. The current study's findings also suggest the need for the support offered to be formalised and provided by trained individuals, potentially those with previous experience of supporting someone treated under the MHA. A formal emotional support service could help to increase its scope and further ensure that carers’ support needs are met.

The importance of sharing information with carers about their family member's/friend's hospital treatment has also been emphasised in previous qualitative literature,^3^ with confidentiality being cited as a key consideration to how much information can be shared. Jankovic et al^3^ described information sharing as a delicate balance between giving carers more information and ensuring patient confidentiality. It has been suggested that general information around patients’ treatment that builds on what carers already know about their family member/friend can be shared without patient confidentiality being broken.^26^ This aligns closely with carers’ views in the current study. It is important that professionals are aware of the type of information they can share with carers without breaching patient confidentiality, and that carers are aware of the type of information they can receive about their family member/friend or reasonably request.

### Strengths and limitations

To our knowledge, this is the first study to explore what support carers would like to receive during their family member's/friend's involuntary hospital admission. Interviews were conducted at three sites in England, which were all markedly different in their population density, diversity and deprivation levels, enhancing the representativeness of the study sample.^27–29^ All interviews were coded by four multidisciplinary researchers, and carers with experience of supporting someone who had been involuntarily admitted to hospital were involved in study development and analysis.

However, there are some limitations. There is potential for selection bias in terms of the carers who access this type of research, with underserved carers being less likely to have access to this type of research. Additionally, the sampling method used in this study (purposive sampling) may limit the generalisability of the findings.^30^ Although efforts were made to recruit a more diverse sample, including reaching out to communities of typically underrepresented groups and approaching personal contacts of lived experience and professional members involved in the study, we received interest mainly from those who were either female or of White British ethnicity, meaning that their voice and preferences are represented more strongly than other groups. Further research is needed to capture the perspectives of more diverse communities. To help capture these perspectives within this field and across other research, further work should be done examining decision-making factors for participating in research across various communities and across a male demographic. All the interviews were conducted online meaning that the rapport generally obtained through face-to-face contact may have been missed. However, online interviews have been found to elicit notable rapport and rich data.^31^ Finally, we did not ask for feedback from participants regarding their transcripts.

### Implications

The current study provides key areas for the provision of support for carers, based on their experiences of the current support offered to them either by the mental health services or through personal contacts. Based on these experiences, the current study's findings show that the support received by carers is unstructured and often either left to chance or informal peer contacts. Key areas of support identified include the need for personal continuity and support from those with lived experience. This could be offered through a formal support programme that provides relevant information and a named contact person, ideally with lived experience. This type of support programme could reduce costs and input required by mental health professionals, who are already under significant strain. Providing support that directly addresses carers’ needs may help to improve their well-being, which could reduce potential long-term costs associated with psychological or physical morbidity in this group. By feeling supported, this type of programme could also lead to carers providing a greater contribution to services than what they already provide.^6,7^ We hope that our study findings can be used to inform co-production in the development of a support programme for carers, ensuring that carers’ experiences are considered and utilised from the outset.

## Data Availability

The data that support the findings of this study are available from the corresponding author, D.G., upon reasonable request.
